# Challenges and advances in measuring sap flow in agriculture and agroforestry: A review with focus on nuclear magnetic resonance

**DOI:** 10.3389/fpls.2022.1036078

**Published:** 2022-11-08

**Authors:** Ritesh Kumar, Mohsen Hosseinzadehtaher, Nathan Hein, Mohammad Shadmand, S. V. Krishna Jagadish, Behzad Ghanbarian

**Affiliations:** ^1^ Department of Agronomy, Kansas State University, Manhattan, KS, United States; ^2^ Department of Electrical & Computer Engineering, University of Illinois, Chicago, IL, United States; ^3^ Department of Plant and Soil Science, Texas Tech University, Lubbock, TX, United States; ^4^ Porous Media Research Lab, Department of Geology, Kansas State University, Manhattan, KS, United States

**Keywords:** magnetic apparatus, nuclear magnetic resonance, plant, sap flow, stem, xylem

## Abstract

Sap flow measurement is one of the most effective methods for quantifying plant water use.A better understanding of sap flow dynamics can aid in more efficient water and crop management, particularly under unpredictable rainfall patterns and water scarcity resulting from climate change. In addition to detecting infected plants, sap flow measurement helps select plant species that could better cope with hotter and drier conditions. There exist multiple methods to measure sap flow including heat balance, dyes and radiolabeled tracers. Heat sensor-based techniques are the most popular and commercially available to study plant hydraulics, even though most of them are invasive and associated with multiple kinds of errors. Heat-based methods are prone to errors due to misalignment of probes and wounding, despite all the advances in this technology. Among existing methods for measuring sap flow, nuclear magnetic resonance (NMR) is an appropriate non-invasive approach. However, there are challenges associated with applications of NMR to measure sap flow in trees or field crops, such as producing homogeneous magnetic field, bulkiness and poor portable nature of the instruments, and operational complexity. Nonetheless, various advances have been recently made that allow the manufacture of portable NMR tools for measuring sap flow in plants. The basic concept of the portal NMR tool is based on an external magnetic field to measure the sap flow and hence advances in magnet types and magnet arrangements (e.g., C-type, U-type, and Halbach magnets) are critical components of NMR-based sap flow measuring tools. Developing a non-invasive, portable and inexpensive NMR tool that can be easily used under field conditions would significantly improve our ability to monitor vegetation responses to environmental change.

## Introduction

Agricultural irrigation accounts for roughly 70% of global freshwater use and in some of the least developed countries, this number can reach upwards of 95% (FAO - SOLAW, https://www.fao.org/3/i7959e/i7959e.pdf). This rate of utilization is unsustainable and in many regions outpaces the natural restoration rate of the recharging aquifers causing a depletion in water resources (FAO – TAWAFSF, https://www.fao.org/home/en). Without improvements in plant water use efficiency or efficient water management practices, the rate of depletion would continue to rise to meet increased demand for agricultural products due to a combined increase in population and demand for global food supply (Hatfield and Prueger, 2015). In addition to these impending demands on the agriculture community, climate variability plays a more significant role in reducing production rates as extreme weather occurs more frequently and with greater intensity (FAO-CC14, https://www.fao.org/3/i5188e/I5188E.pdf). Recent studies focused on extreme events have shown that food production systems and water supplies are exceptionally vulnerable to climatic variability (FAO-CC14). More specifically, climatic variability is shown to negatively affect water availability and the ability to meet irrigation demands (Wada et al., 2013). The combination of increased population, demand for food and feed, and limited access to water would result in an increased demand for irrigation, which would ultimately deplete the natural groundwater reserves and threaten future agricultural productivity (Portmann et al., 2013; Meixner et al., 2016).

Limited water available scenarios would result in the decrease of amount of water in plants resulting in drought stress. Drought stress causes reduced cell size, photosynthesis, altered transcript abundances of transcription factor families, decreased turgor pressure and crop yields (Samarah, 2005; Kaya et al., 2006; Estrada-Campuzano et al., 2008; Jiao et al., 2021). Crop yield may decrease by 30% to 90% in fields under drought, although it varies from one crop species to another and depends on the intensity and the duration of stress (Hussain et al., 2019). Cereals are a significant part of our food supply, yet their agricultural production is heavily impacted by drought stress (Elliott et al., 2014; Kadam et al., 2014). The loss of agricultural yield caused by drought occurs in virtually every climatic region in the world (Daryanto et al., 2016). Since 1983, drought has reduced the overall yield of maize (*Zea mays* L.), rice (*Oryza sativa* L.), soybeans (*Glycine max* L.) and wheat (*Triticum aestivum* L.) in nearly 75 percent of all harvested areas (Kim et al., 2019). Similarly, using a modelling approach, studies have found that from 1980 to 2015 drought reduces yields of different crops like wheat, rice, soybean, maize by 21 - 28%, 25%, 21% and 39% respectively (Daryanto et al., 2016; Zhang et al., 2018).

Genetic background, environment, and management interact to determine crop yield and must be optimized to achieve the maximum yield potential. Recent studies have revealed that agriculture could expand northward due to the warming of high latitude regions and increasing food demand (King et al., 2018). Maize, oat (Avena sativa L.), and wheat cropping areas in Europe are likely to expand northward due to climatic warming (Elsgaard et al., 2012). Warming temperature in China has led to both westward and northward expansion of winter wheat (Yang et al., 2011) and maize (Hu et al., 2019). Due to the northward shift of the agricultural climate zone, many of the newly gained areas are linked to changes in climatic water balances. This is an important factor to consider in future land-use and management decisions (King et al., 2018). With a better understanding of plant water relations and soil moisture content, these agricultural land shifts can be accomplished more efficiently with more appropriate genotypic selections.

It is essential to understand mechanisms of plant water in response to soil and atmospheric drought. To effectively study plant water relations, the environmental and plant conditions must be accurately and rapidly measured and recorded. The study of this movement of water from soil to plant and to atmosphere is referred as soil-plant-atmosphere continuum (SPAC), which has become a dominant framework for understanding plant water movement (Deng et al., 2017). The most critical components of the SPAC are plant water potential, soil moisture content, plant uptake, evapotranspiration, and dynamics of xylem transport (Vicente et al., 2003; Fayer and Gee, 2004; Vidana Gamage et al., 2021). In plants, sap flow in the stem is used as an indicator of transpiration and water usage. Transpiration may be measured using the gas exchange system method. The rate of transpiration can be used to quantify the amount of water used by the plant and can lead to a greater understanding of plant water hydraulics under varying conditions (Steppe et al., 2015). Most popular techniques for measuring sap flow are based on heat balance and thermal dissipation. However, these are invasive techniques that are prone to multiple types of errors, such as misalignment of probes (Ren et al., 2017). Among technologies available to measure sap flow, nuclear magnetic resonance (NMR) is a non-invasive method, and several attempts have been made to manufacture portable and accurate NMR tools for sap flow measurement (Windt and Blümler, 2015).

Although the sap flow measurement literature is vast and extensive, it still lacks in a comprehensive review that addresses most recent advances and challenges. In this study, we particularly focus on nuclear magnetic resonance as a promising, non-destructive, and portable method for sap flow measurements. We overview mechanisms of transpiration and sap flow from biological viewpoint, address the importance of sap flow measurement in agriculture, review methods for quantifying sap flow and their advantages and limitations, and discuss portable nuclear magnetic resonance NMR system in measuring sap flow.

## Plant hydraulics: The ascent of water

**Figure 1:**
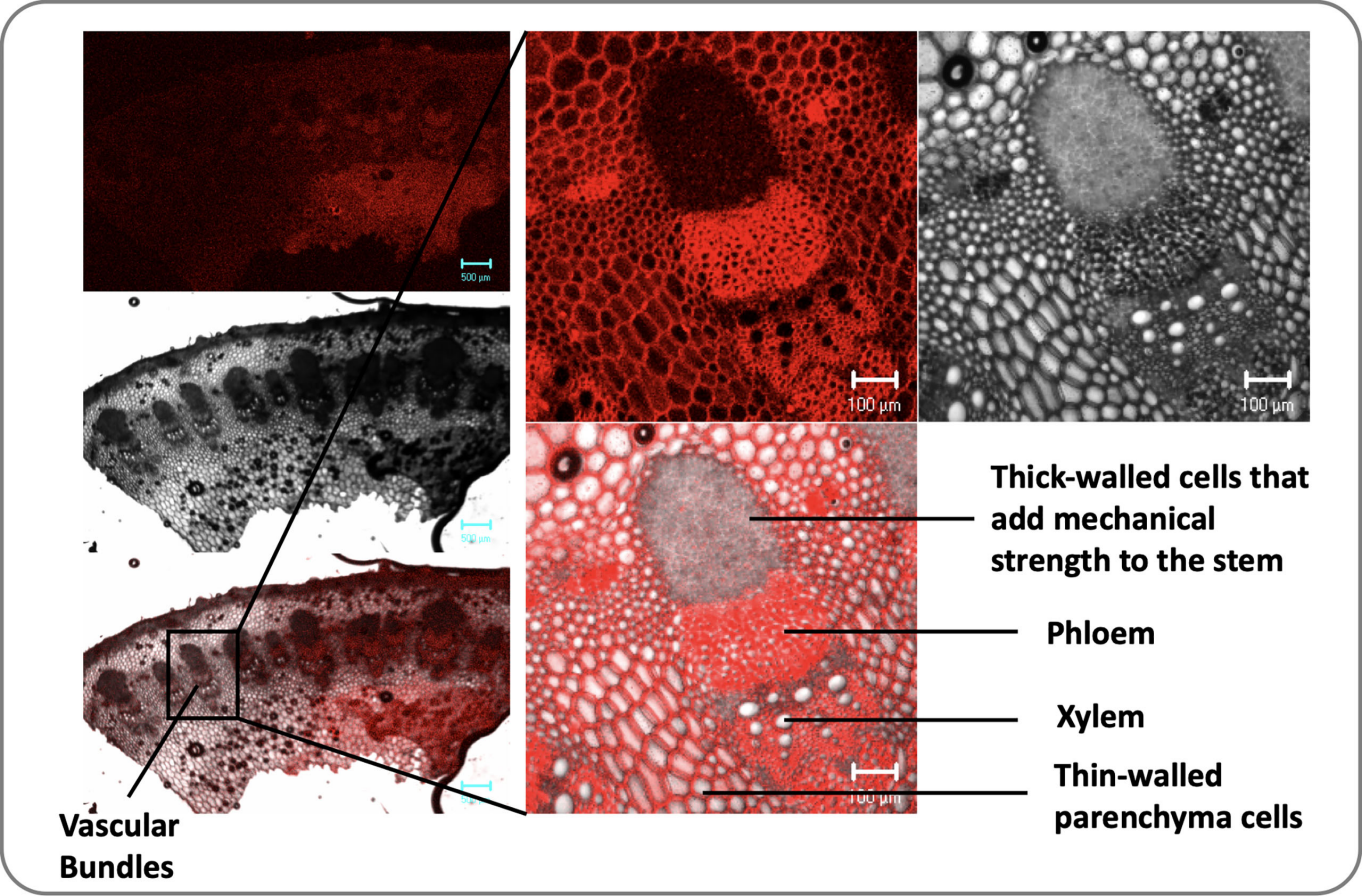
Transverse sections of the dicot stem (sunflower) illustrate vascular bundles’ arrangement. Vascular bundles are tubelike tissues that flow through plants, transporting different essential substances including water. Each vascular bundle consists of a one xylem and one phloem and there are several vascular bundles in the plant stem.

**Figure 2:**
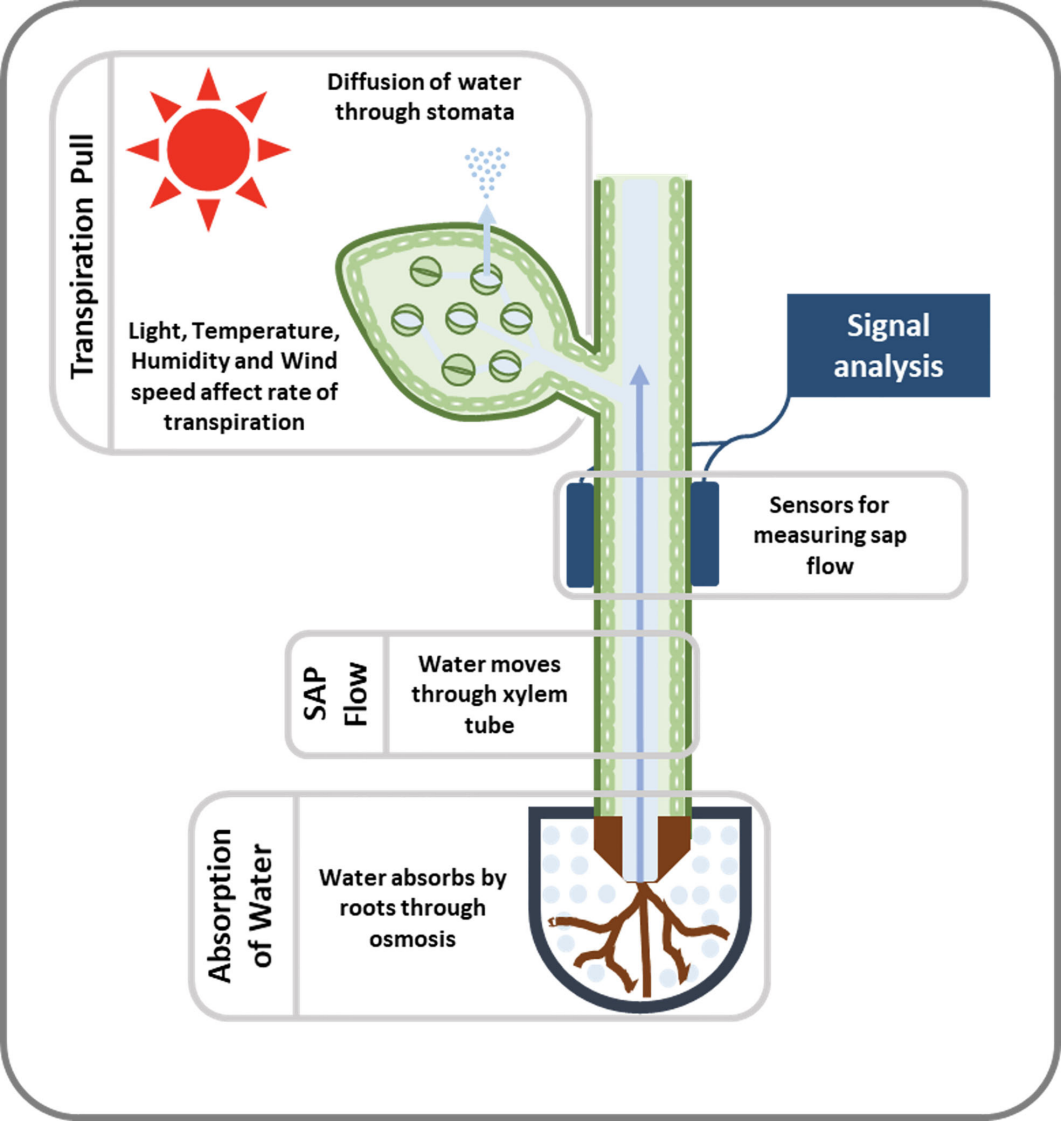
An illustration of the movement and measurement of sap flow. When leaves transpire, they generate negative pressure or suction that causes water to move upward. Sensing devices (used to measure sap flow) are attached to the stem of the plant or tree and detect this movement of SAP. To determine SAP velocity or SAP flux, the signal received from the sensors is detected and decoded.

Plants have a complex transport system consisting of vascular bundles extending from roots to leaves and consisting of xylem and phloem (**Figure 1**). The most widely accepted approach for the ascent of sap is the transpiration cohesion-tension theory purposed by Dixon and Joly (1894). According to this theory, the upward movement of water is due to the negative pressure (suction) created by leaves during transpiration (driven by atmospheric pull) causing tension on the continuous water column in xylem (Dixon and Joly, 1894). Before entering the xylem, water is taken up by roots and must cross various cell layers, which act as a filtration system (Fiscus, 2015). Water travels through root epidermis and endodermis *via* nonexclusive pathways, such as the apoplastic, symplastic, and transcellular to reach xylem (Frensch and Hsiao, 1993). A network of non-living cells makes up the xylem tissue, which transports water from roots to leaves (Tyree and Zimmermann, 2002). In transpiration water passes through spongy mesophyll cells in leaves, it enters pores called stomata and through them into the atmosphere as water vapor (**Figure 2**).

In addition to transporting water, xylem sap carries inorganic nutrients from soil to other parts of a plant. Xylem sap contains sodium, calcium, potassium, magnesium, phosphorus, sulfur, boron, zinc, and copper (Pate et al., 1998; Jiang et al., 2004). Xylem sap also carries amino acids, proteins, plant hormones, and their metabolites (Bano et al., 1994; Bahrun et al., 2002; Kehr et al., 2005; Alvarez et al., 2006; Krishnan et al., 2011). However, the exact composition of xylem sap can vary significantly due to temporal, phenological, and environmental changes (Goodger et al., 2005; Alvarez et al., 2008; Krishnan et al., 2011).

Initially, it was believed that stomata closed at night and did not account for night-time water loss. However, several studies showed that many plants have significant night-time stomatal conductance, and 5-30% of water loss occurs due to night-time transpiration (Assaf and Zieslin, 1996; Oren et al., 2001). More specifically, Snyder et al. (2003) found that 11 of 17 different species of C3 and C4 plants used in their study showed night-time stomatal conductance. Positive effects of night-time transpiration include the ability to transfer nutrients to distal areas as well as prevent the accumulation of excessive leaf turgor pressure in the plants with high leaf osmotic potential (Donovan et al., 2001; Rohula et al., 2014).

## Importance of sap flow measurement in trees and plants

Agricultural water management is one of the most important applications of sap flow measurement in a production environment. Irrigation management is critical in areas with insufficient water supplies and in drylands where water resource assessment and management are necessary for effective production as rainfall generally falls short of evapotranspiration demand (Koch and Missimer, 2016). The use of sap flow sensors helps irrigation management and timing as they provide accurate data on plants water demand (Jones, 2004; Esteves et al., 2015). Furthermore, such sensors are useful for managing irrigation based on soil and weather conditions. Based on sap flow measurement researchers have designed devices, which can calculate irrigation dose to replenish crop water needs (Fernández et al., 2008b; Fernández et al., 2008a; Ballester et al., 2013). Some of these devices can also be remotely controlled and provide daily irrigation amounts based on irrigation protocols (Fernández et al., 2008b).

Sap flow measurements for different species can provide insight regarding which one might be best suited under extreme conditions. For instance, by analyzing three Eucalyptus species, *E. cladocalyx*, *E. melliodora* and *E. polybractea*, it was found that *E. cladocalyx* tolerated saline soils, hotter and drier weather patterns more than the other two species (Doronila and Forster, 2015). In this study the heat ratio method was used to determine sap flow and tree water content. Measures of sap flow in these three eucalyptus trees (grown in the high saline condition) were made over 15 days including 3 days with temperature higher than 40°C. The mean ( ± S.D.) tree sap flux density measured per day was 287.8 cm^3^cm^−2^day^−1^ ( ± 87.2) for *E. cladocalyx*, 82.2 cm^3^cm^−2^day^−1^ ( ± 45.7) for *E. melliodora* and 81.5 cm^3^cm^−2^day^−1^ ( ± 42.5) for *E. polybractea*. A similar study was conducted on tropical tree saplings of *Pterocarpus indicus*, *Lagerstroemia speciosa*, and *Swietenia macrophylla* across wet and dry seasons in 2017–2018 by Andriyas et al. (2021), who estimated canopy conductance and transpiration from sap flux density over wet and dry seasons. They found that *L. speciosa* may perform better than *S. macrophylla* and *P. indicus* under future variable climatic conditions by balancing stomatal closure (Andriyas et al., 2021). In another study, (Suárez et al., 2021) examined diurnal patterns in sap flow from cacao trees grown in three types of agroforestry systems with differing levels of solar radiation. Based on the differences between cacao sap velocity, they developed a model to estimate transpiration and water use in cacao trees as well as to manage cacao agroforestry systems in the Amazon Rainforest. Further, in another study researchers showed the use of measuring sap flow in cocoa planting with the goal of increasing biodiversity should take into consideration the direction of land use and biodiversity transitions ( (Niether et al., 2020; Maney et al., 2022).

Another major application of sap flow measurement is to detect diseased plants (Deng et al., 2015; Roper et al., 2021). The vascular system is essential to plant health, and its damage can have an irreversible effect. Vascular occlusions, such as the formation of tyloses and gums, or the destruction of vessels by microbes, can disrupt hydraulic flux (Sun et al., 2013). Sap flow measurements were made on pine trees inoculated with *Ophiostoma clavigerum* i.e., a blue-stain fungus (Yamaoka et al., 1990). In this study heat pulse velocity (HPV) sensors were used to monitor sap flow daily for four weeks. The fungus (*Ophiostoma clavigerum*) was associated with mountain pine beetle (*Dendroctonus ponderosae*) and this pine beetle impaired tree sap flow (Amman and Schmitz, 1988) in lodgepole pine tree. A similar study revealed that sap flow in Norway spruce (*Picea abies*) ceased within 4–6 weeks after inoculation with a virulent fungus Ceratocystis polonica, an associate of the spruce bark beetle *Ips typographus* (Kirisits and Offenthaler, 2002). Sap flow measurements were used to detect reduced flux for tamarisk trees (*Tamarix* spp.) during defoliation by the salted cedar leaf beetle (*Diorhabda carinulata*) (Hultine et al., 2010). McElrone et al. (2010) showed that mature walnut trees affected by apoplexy disorder had hydraulic failure with the formation of tyloses in the Central Valley of California. More recently, a study by Ouadi et al. (2021) showed that sap flow dynamic, which is correlated with white-rot necrosis levels, is a useful indicator to predict plants suffering from Esca-grapevine disease.

In Canada and North America, xylem sap of several species within the genus Acer is extracted to produce maple syrup, a non-timber forest product of great value (Doner, 2003). Climate change has had a detrimental effect on maple syrup production (Matthews and Iverson, 2017). An analysis carried out at six different geographic sites in North America for over 2–6 years showed that sap collection advances by 4.3 days for every 1°C increase in March mean temperature (Rapp et al., 2019). Additionally, by 2100, the area of maximum sap flow is expected to shift northward by 400 km (Rapp et al., 2019). In that study, sap flow measurements were used as an important tool in revealing that the changing climate conditions affected the number of freeze/thaw cycles, sap collection days, and total sap amount.

## Techniques used for quantifying sap flow

To determine the amount of sap flowing through a surface per unit time, proposed methods measure either flow rate (gh^-1^) or flux density (cm^3^cm^-2^h^-1^) (Vandegehuchte and Steppe, 2012). Different approaches available to measuring sap flow are based on thermodynamics, electrodynamics, or magneto-dynamics concepts. Of the available methods, those based on thermodynamics are among the most widely used (Čermák et al., 2004). Many thermodynamic-based sap flow sensors are available commercially. Thermodynamic techniques for sap flow measurement can be categorized into three major groups: heat balance (HB), thermal dissipation (TD), and heat pulse velocity (HPV) (**Table 1**). The HB method involves circling stem with a thin flexible heater and surrounding it with foam insulation allowing a known amount of heat to be applied (Sakuratani, 1981). This approach considers both conduction and convection losses in stem, heat loss due to the mass flow of water in stem are estimated (Sakuratani and Abe, 1985). In the TD method, two needles mounted with a thermocouple are inserted radially in the tree stem 10cm apart to detect convective heat transfer (heat transported with the sap stream). In the HPV method, a pulse of heat is injected into xylem, and its passage is detected further along the flow direction. By measuring the pulse travel time, the flow velocity is estimated (Forster, 2017). These heat-based methods are majorly used for sap flow measurement more than any other methods. The advantage of using these heat-based methods for sap flow measurement is that they are comparatively easy to use than other methods, portable, and most of them are commercially available. But there are many major drawbacks associated with these methods. The heat-based methods involve inserting probes, which causes wounding and mechanical damage to the plants. Misalignment of probes may also result in substantial uncertainties in sap flow measurements. For example, the study by Burgess et al. (2001) demonstrated that a 1-mm difference in probe spacing in the compensation heat pulse method (CHPM) would render 20% error in the heat pulse velocity. In another study on Eucalptus marginata, Bleby et al. (2004) found a 2-mm error in spacing caused a 100% error in sap velocity determination. Moreover, installing these components (like probes) leads to the species-specific wounding responses, resulting in accounted and unaccounted errors in measurements (Bush et al., 2010; Wullschleger et al., 2011). In plants, a wound due to a drill hole generally induces tyloses formations (Barnett, 2004) that cause significant reductions in hydraulic conductance and sap flow (Sun et al., 2006). Heat pulse methods use a default value of thermal diffusivity (heat tra\nsfer rate). However, evidence show that the value of thermal diffusivity changes at a faster rate than the period of measurement resulting in an overestimation of sap flow (Looker et al., 2016). Other parameters, such as stem moisture content and sapwood radial variability, can also contribute to errors during sap flow measurement and produce unreliable results. A comparative study showed that the actual flux density was underestimated by 35, 46, and 60% using the heat pulse velocity, heat field deformation, and thermal dissipation respectively (Steppe et al., 2010).

**Table 1:** Different heat-sensing methods/techniques used for sap flow measurement.

No.	Method Names	Family	Advantages	Disadvantages	Invasive/Non-invasive	Purposed by/ References	Available from
**1**	Stem heat balance (SHB)	Heat balance methods	1. Stem and roots 3. size between 2 to 125 mm 3. optimal time of irrigation	1. Large error when sap flow is low 2. Can kill trunk when not installed properly	Non-invasive	(Vieweg and Ziegler, 1960)	Dynamax Inc, EMS Brno and ICT International
**2**	Trunk sector heat balance (TSHB)				Invasive	(Čermák et al., 1973; Tatarinov et al., 2005; Ktjčera et al., 2008)	EMS Brno and ICT International
**3**	Thermal dissipation (TD)	Thermal dissipation methods	Provides continuous measurement	1. Lack of measurement accuracy 2. High energy consumption 3. calibration is difficult	Invasive	(Vieweg and Ziegler, 1960; Ittner, 1968; Balek and Pavlík, 1977; Granier, 1985)	Dynamax Inc, EKOMATIK and ICT International and UPGmbH
**4**	Heat field deformation (HFD) method				Invasive	(Nadezhdina, 1999)	ICT International
**5**	Transient thermal dissipation (TTD) method				Invasive	(Do et al., 2011)	
**6**	Compensation heat-pulse (CHP) method	Heat pulse velocity methods	1. Measures flow volume and orientation (low, zero and reverse flow) 2. Stem size > 10mm 3. Stem and roots 4. Low power usage	1. Wound corrections need to be applied 2. Not suitable for herbaceous plants	Invasive	(Huber, 1932)	
**7**	Cohen’s heat-pulse method, or T-max method				Invasive	(Huber, 1932; Marshall, 1958; Cohen et al., 1981)	Advanced Measurements and Controls Inc and Tranzflo NZ Ltd
**8**	Heat-pulse velocity (HPV)				Invasive	Green, 1998	ICT International
**9**	Heat ratio (HR) method				Invasive	(Burgess et al., 2001)	
**10**	Sapflow+ method				Invasive	(Vandegehuchte and Steppe, 2013)	

To reduce errors in sap flux density measurements, one may use a single probe containing three-temperature sensors that can autocorrect misalignment (Ren et al., 2017). With probe spacing corrections applied in this study, the root mean squared error (RMSE) values for CHP, HR, and T-max methods decreased from 4.82, 3.09, and 2.73, respectively, to 3.1, 2.18, and 0.82 (Ren et al., 2017). In addition, a sap flow sensor design with all sensor components on a single integrated circuit board helps minimizing misalignment errors (Jones et al., 2020). There are still other factors needed to be eliminated in future research, such as differences in axial versus tangential thermal properties, distance-dependent thermal properties of trees, and wounds that may cause errors in sap flow velocity.

In the literature, several non-invasive techniques to measure sap flow have been proposed. Helfter et al. (2007) used a non-invasive pulsed laser-based technique to monitor xylem and phloem flow in woody plants. In their method, infrared lasers produced heat pulses were used to detect sap flow. The laser source produces heat on the stem surface, and an infrared camera monitors the heat propagation externally, and finally using thermometric data heat pulse velocity is calculated. Another non-invasive way is measuring sap flow velocity using different dyes e.g., fuchsin, safranin, and eosin. These are one of the oldest methods to visualize water-conducting pathways. Xylem specific dyes like acid fuchsin and crystal violet were used to study conductive area and flow pathways from roots to shoots of 20-year-old Thompson Seedless and 8-year-old Chardonnay grapevines (Harris, 1961; McElrone et al., 2021). Using different dyes sapwood of various conifers was studied including *Pseudotsuga taxifolia* (Poir.) Britt., *Pinus nigra* Arnold, and *P. radiata* D. Don. Acid fuchsin uniformly stained the late wood because it diffused easily, but safranin did not, and it remained uncolored (Harris, 1961). Acid fuchsin dye is also used to study patterns of water movement in four species of gymnosperms and seven of angiosperms (Kozlowski and Winget, 1963). Likewise, eosin dye helps measure the conducting capacity of lateral veins of the wheat leaf (Altus et al., 1985). A major disadvantage of using dyes is that it is not known whether they would travel as far as the sap flow before they deposit on the vessel walls (Marshall, 1958). Furthermore, dyes would never indicate variation in speed along a dyed path but only show the maximum speed along such a path (Marshall, 1958).

Researchers have used a variety of methods based on heat transport (Marshall, 1958), nuclear magnetic resonance microimaging (Köckenberger et al., 1997), magnetic resonance imaging (Homan et al., 2007), optical techniques (Helfter et al., 2007), particle-traced X-ray imaging (Ahn et al., 2013), acoustic emission technique (Zweifel and Zeugin, 2008), and high-resolution computed tomography (Brodersen et al., 2019) to determine sap flow in plants (Alarcón et al., 2000). Lee and Kim (2008) explored plant water-refilling processes using synchrotron X-ray microimaging. Then, Kim and Lee (2010) used non-destructive synchrotron X-ray imaging to monitor sap flow through the xylem vessels of rice leaves. Positron emission tomography (PET) scanners is advancing as a powerful functional imaging technique to decipher *in vivo* the function of xylem water flow (with 15O or 18F), phloem sugar flow (with 11C or 18F) (Hubeau and Steppe, 2015). In a recent study, plant PET scanners were used to study phloem vulnerability under hotter drought conditions in Populus tremula (Hubeau and Steppe, 2015). A magnetic resonance imaging (MRI) scan of a living plant stem can also be used to determine the location of the xylem vessels and understand their status. Using this approach, if these vessels were filled with liquid, the velocity, and direction of the movement of this liquid, and metabolites present in it can be identified (Windt et al., 2009; van As et al., 2009).

Magnetic resonance imaging (MRI) is most probably the only non-invasive techniques used to measure both xylem and phloem sap flow. However, the currently available equipment are bulky, expensive, and not suited for field measurements. Instead, various intrusive techniques use heat to trace sap flow in the field. Due to probe implantation, these methods disrupt the flow being measured and are unsuitable for phloem monitoring due to the fragility of the tissue.

## Nuclear magnetic resonance for sap flow measurements

Nuclear magnetic resonance (NMR) is a phenomenon when a strong magnetic field and the nucleus-based phenomenon is basically related to the absorption and re-emission of electromagnetic wave radiation. This phenomenon was introduced in 1938 (Rabi et al., 1938; Rabi et al., 1939). In the early 1940s, Felix Bloch and Edward Purcell independently developed the NMR method for measuring nuclear magnetic moments, for which they shared the Nobel Prize in 1952. NMR applications developed rapidly over the next 50 years. Nuclei with odd numbers of protons and neutrons have non-zero spins, making them act as tiny bar magnets. Their orientation is normally random and there is no net magnetic field, so they cannot generate an NMR signal. In NMR, when these nuclei are subjected to an external strong and homogenous magnetic field, nuclei spins are orientated along the magnetic field direction; some in parallel (low energy) and some others in antiparallel directions (high energy). This results in a net magnetic moment generated which is aligned with the external magnetic field. This net magnetization can be pushed away from the alignment using a second magnetic field applied perpendicular to the external magnetic field with an excitation coil. Net magnetization returns to the external magnetic field when this second field is turned off, generating a radiofrequency signal which is detected at the detector coil. This signal forms the basis of NMR imaging and spectroscopy.

The energy difference between the two states of nuclei spin under an external strong magnetic field is very small and corresponds to a range of frequency of the EM spectrum. A short duration radio frequency signal is used to flip the nuclear spin from the spin-aligned state of low energy to the spin-opposed state of high energy. Thereafter, the charged nucleus will exhibit processional motion with a specific frequency, known as the Larmor frequency. The actual magnetic field created at a nucleus is also dependent on the electrons surrounding it, which creates an ionic environment around it which leads to a variation in the frequency of resonating particles, known as chemical shift. This change magnetic field is known as shielding. Energy is absorbed by the nuclei during the transition from lower to higher energy levels and released during the opposite process. The process of returning to the original energy state is known as relaxation. The two most common types of relaxation are spin lattice relaxation (T1) and spin relaxation (T2). T1 relaxation, also known as spin lattice or longitudinal relaxation is the time constant used to describe when ~63% of the magnetization has recovered to equilibrium. T2 relaxation involves energy transfer between interacting spins *via* dipole and exchange interactions. Spin-spin relaxation energy is transferred to a neighboring nucleus. Now there are two NMR methods continuous and pulsed method. In continuous wave NMR method, a sample in a static magnetic field is applied by RF perpendicular to the sample and when an excitation frequency is reached, oscillation is observed at the detector. In the pulsed case, one applies a sequence of RF pulses called π-pulses (180-degree pulses) or π/2-pulses (90-degree pulses) and looks for “free induction decay” and “spin echoes”. Use of Pulse NMR experiments are most popular these days.

**Figure 3:**
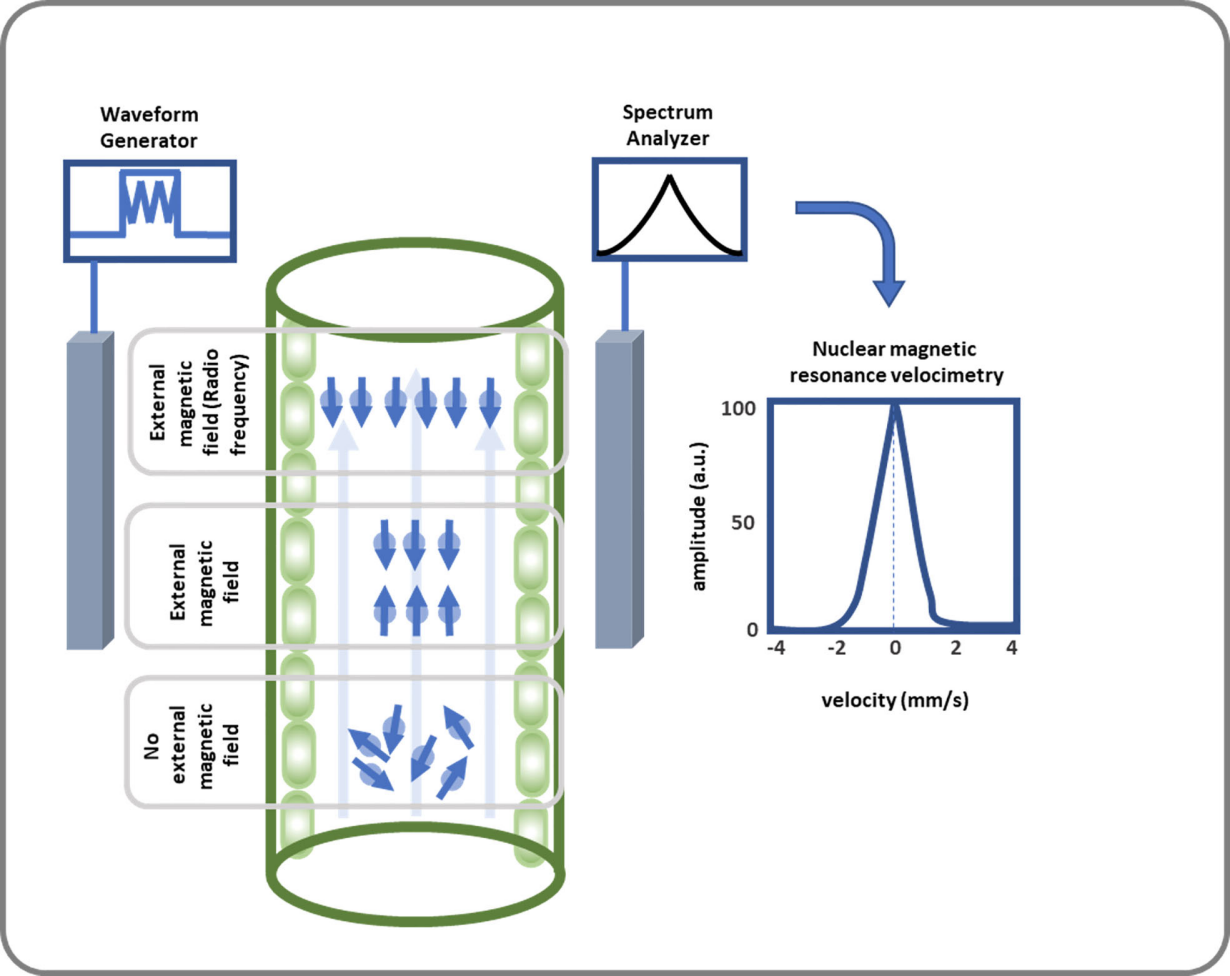
This diagram illustrates how SAP flow is measured using NMR. SAP flow measurement using NMR includes a strong magnetic field where 1H nucleus gains a quantum mechanical property callspin at. Then an NMR signal is created by applying RF energy equal to the Larmor frequency, which leads to nuclei transitioning from their groan und to excited state, and spins being oriented oppositely or antiparallelly. After receiving, amplifying, and further analyzing the signal, the volumetric flow rate of the xylem and phloem is calculated.

Capturing all details of these reaction can be conducted by accurate measurement systems. The captured waveforms which are known as NMR signals, release fundamental features regarding the understudy materials; see **Figure 3** (Callaghan et al., 1991). Concept of NMR has been applied in many research areas e.g., in plant studies to determine moisture in apple (Shaw and Elsken, 1956), unfrozen water in winter cereals at subfreezing temperatures (Gusta et al., 1975), and xylem embolisms in pine wilt disease (Umebayashi et al., 2011). One advantage of NMR is that it can spatially resolve both static and dynamic parameters and generate data in a non-destructive manner from the interior of a sample.

NMR can be used in different applications where non-destructive measurements are required. NMR imaging is also widely used to measure sap flow (**Table 2**). van As and Schaafsma (1984) investigated water flow through the stem of an intact cucumber plant using pulsed NMR. In their study, the NMR signal due to high stationary water was cancelled out so that it doesn’t interfere in the detection of flowing water. This made it an important application for measuring water flow to plants with high stationary water content. Using NMR microimaging, Köckenberger et al. (1997) measured the phloem and xylem water flow in castor bean seedlings. This study demonstrated for the first time that water is internally circulated between phloem and xylem. Even in absence of significant transpiration or evaporation, there is water maintenance through this water circulation. Fast gradient echo sequence (FLASH) imaging of flow velocities, as a fast-imaging NMR method, was introduced by Rokitta et al. (1999). Their method achieved a six-fold reduction in imaging time while maintaining a similar signal-to-noise ratio to previous flow NMR imaging techniques. Scheenen et al. (2000) developed the pulsed field gradient (PFG) NMR technique combined with turbo spin-echo imaging, which resulted in an accurate and spatially resolved sap flow measurement. Windt et al. (2006) used NMR imaging for diurnal dynamics study of phloem and xylem transport in poplar (Populus spp), castor bean (Ricinus communis), tomato (Solanum lycopersicum) and tobacco (Nicotiana tabacum). They found large diurnal variation in xylem flux and small or no change in phloem flux.

**Table 2:** Different studies used NMR and other imaging methods to study sap flow measurement in plants and trees.

**No.**	**Methods used**	**Problem addressed**	**Year**	**References**
**1**	Pulsed NMR	Water flows through an intact cucumber plant (*Cucumis-sativus L.*)	1984	(van As and Schaafsma, 1984)
**2**	Portable NMR	Xylem sap stream and tissue water content in a cucumber plant	1994	(van As et al., 1994)
**3**	NMR imaging	Water transport in chrysanthemum flower stem segment	2000	(Scheenen et al., 2000)
**4**	NMR	Water flow velocity and solute transport in xylem and phloem of adult plants of *Ricinus communis*	2001	(Peuke et al., 2001)
**5**	NMR	Phloem solute and water transport at the hypocotyl of Ricinus communis	2005	(Peuke et al., 2006)
**6**	MRI	Phloem and xylem flow characteristics and dynamics in poplar, castor bean, tomato and tobacco	2006	(Windt et al., 2006)
**7**	MRI	Xylem and phloem flow in an intact small cherry tree	2007	(Homan et al., 2007)
**8**	MRI	Dynamics in Long-Distance Sap Flow and Flow-Conducting Surface Area in an intact cucumber (Cucumis sativus) plant	2007	(Scheenen et al., 2007)
**10**	MRI	Monitor xylem cavitation, refilling, and functionality in maize	2009	(Kaufmann et al., 2009)
**12**	NMR flow imaging	Xylem and phloem flow imaging study in tomato truss	2009	(Windt et al., 2009)
**14**	1H MRI	Changes in water content and distribution in *Quercus ilex* leaves during progressive drought	2010	(Sardans et al., 2010)
**15**	Portable NMR-CUFF	Xylem sap flow in a young poplar tree	2011	(Windt et al., 2011)
**16**	Mobile MRI system	microscopic water flow in the Japanese pear tree (*Pyrus pyrifolia* Nakai, Kosui)	2011	(Kimura et al., 2011)
**17**	MRI	Stem water content to stem diameter variations in Oak (*Quercus robur L.*) tree	2012	(de Schepper et al., 2012)
**19**	Low-Field NMR and Neutron Imaging	Observation of drought response of juniper (*Juniperus monosperma*) and pinon pine (*Pinus edulis*)	2016	(Malone et al., 2016)
**20**	MRI system	In situ MRI-flow measurements for a tree living in the outdoors	2016	(Nagata et al., 2016)
**22**	MRI	Xylem and phloem sap flow in an outdoor zelkova tree	2020	(Terada et al., 2020)

Using NMR flow imaging, Windt et al. (2009) demonstrated that, in a developing tomato truss, 75% of net influx into a fruit occurred through the xylem and the remaining 25% *via* the perimedullary region, which includes both phloem and xylem. Using NMR methodology, the moisture content in wood samples could be instantly determined from the mass and amplitude of free-induction-decay (FID) signals and found this methodology to be more precise and reliable than the gravimetric methods regardless of wood species (Merela et al., 2009). Furthermore, NMR imaging can determine the level of hydraulic conductivity in detached stems. Using MRI, de Schepper et al. (2012) showed that elastic bark tissues contribute most to the daily depletion of internal stem water storage. In another study, low field NMR and neutron imaging were used to deduce aspen tree (Populus tremuloides) drought response (Malone et al., 2016). NMR imaging was also used for grape (Vitis vinifera) stems to show that basal leaves were more prone to embolism than apical leaves and these leaves shed to prevent water loss and protect the hydraulic integrity of the plant (Hochberg et al., 2017).

The variety of possible applications of NMR makes it an extremely useful tool to understand plant water relations. However, experiments and applications are currently limited to solely lab-based measurements. To take full advantage of the potential applications of NMR, the technology must be advanced and integrated into a portable system which can be taken to the field condition. In the following, we address current challenges with designing a portable NMR tool for sap flow measurements in plants.

## Challenges in designing portable NMR for sap flow measurement

**Figure 4:**
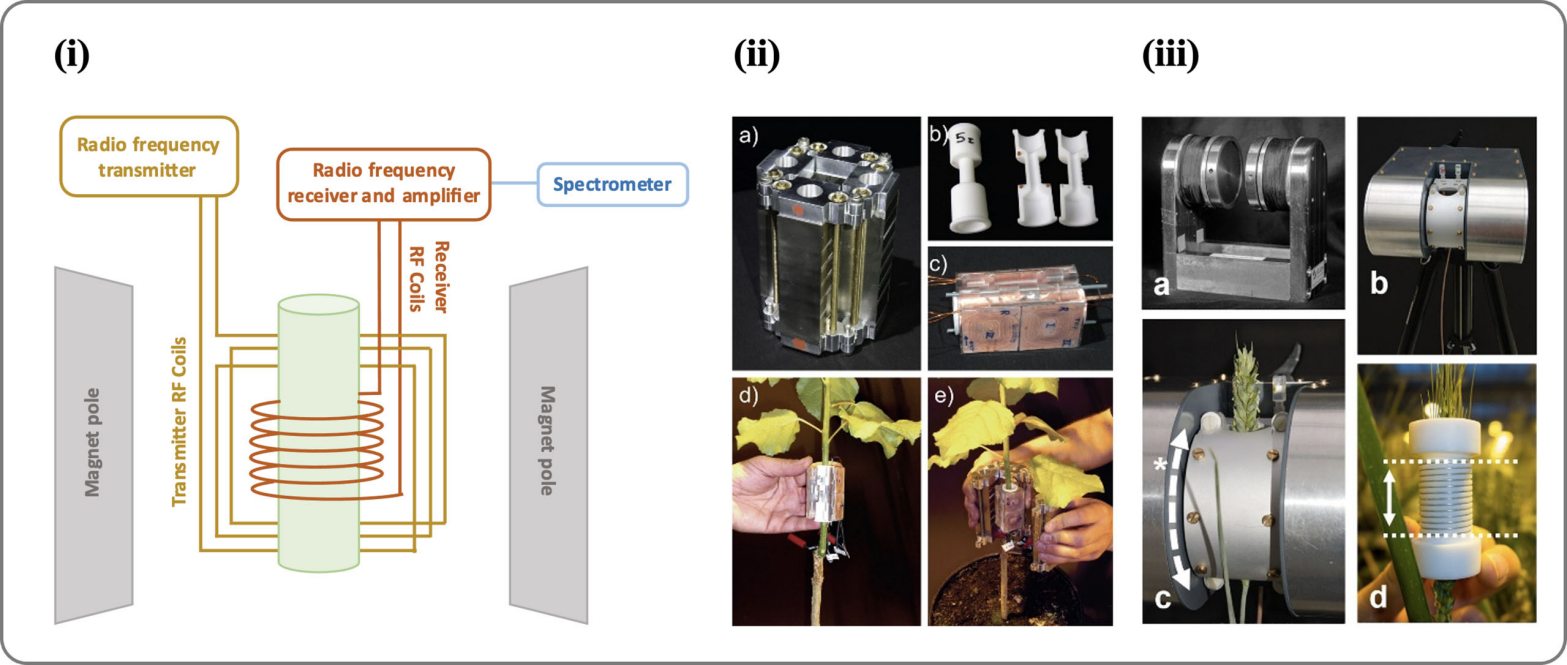
**(i)** An image showing the different parts of an NMR spectrometer. **(ii)** Portable NMR CUFF magnet system (source: Windt et al., 2011). An NMR-CUFF prototype, consisting of a Halbach magnet, is shown in this image; **(A)** shown is the template for an RF-coil; **(B)** the hinged, plane-parallax gradient system assembly with an RF-coil inside; **(C)** mounting the RF coils; and **(D)** enclosing the plant stem with the NMR-CUFF. **(iii)** Mobile NMR Mouse sensor made from a C-shaped magnet (source: Windt et al., 2021). An image of the magnet **(A)** shows a permanent C-shaped magnet, **(B)** an insulating housing or adjustable stand, **(C)** a probe housing that can be rotated around the magnet poles (*) to the sample angle, and **(D)** solenoidal RF coils with dotted lines indicating their sensitive volume (25 mm). For more details see Windt et al., 2021.

NMR demonstrates promising results as a tool for sap flow measurement (Windt et al., 2011). A typical NMR spectrometer system consists of the magnets (to generate an external magnetic field), lock (used to control magnetic field in the sample so that the resonance frequencies do not drift), shim coils (to adjust the homogeneity of a magnetic field), gradient coils (used to produce deliberate variations in the main magnetic field B0), RF coils (to generate the RF magnetic field that excites the nuclear spins, and to detect the MR signal), and a spectrometer (a receiver which detects the magnetic resonance signal) (**Figure 4i**). NMR imaging system can measure the presence and movement of water quantitatively and non-invasively. However, many restraints like complexity and bulkiness in the NMR instrument limit its applicability to field studies. There is a major limitation with NMR in plant sciences due to the lack of small-scale and portable NMR tool that can be used in greenhouses and field conditions. Moreover, these methods generally involve complicated procedures and data processing techniques (Windt et al., 2021; Meixner et al., 2021).

### Homogeneity and strength of magnetic field

According to the main purposes of the NMR technology, providing a strong and homogenous static magnetic field is essential to have a better observation of the transition in nuclear spin. It is worth mentioning that there is a direct correlation between the Larmor frequency, the spin bearing nucleus and the static magnetic field strength. Also, by intensifying the constant magnetic field in the test region, contrast to noise ratio could be improved considerably (Okada et al., 2005). Halbach arrays provide a homogenous and strong magnetic field (Raich and Blümler, 2004), a significant characteristic for NMR-based imaging and velocimetry applications. This is due to a uniform flux that is generated based on the orientation of segmented magnets in the Halbach arrays (Halbach, 1980). In other magnet structures, such as north-south magnets, extra iron plates are commonly needed to be added on exterior face of the ring to minimize the leakage flux and have stronger magnetic field within the ring. The challenge here will be resulting less homogenous magnetic field due to the impact of iron plate of uniformity of flux. In Halbach magnet structures, however, this extra iron plate is not required due to the orientation of segmented magnets, which results in negligible leakage flux. This makes the Halbach magnet-based tool lighter with more homogenous magnetic field within the ring compared to other magnet types. However, designing NMR with Halbach arrays has some challenges as well. For instance, multi-direction pulling forces may be strong enough that an openable mechanical structure could not be released easily during a test cycle. Thus, it is essential to provide an appropriate design to lessen consequences of the existing pulling force in the NMR (Li et al., 2021). The mechanical structure should be in a way that the Halbach rings can be opened with minimal forces required, which can be simulated using finite element analysis tools. It should be mentioned that the excessive extent of pulling force can progressively misalign the finalized NMR mechanical structure, and such a misalignment can demote the level of magnetic field homogeneity (Koch et al., 2009).

Moreover, error in the production process of magnetic parts is another challenge for NMR design. This issue has some adverse effects on the homogeneity of magnetic fields which can be observed by the electromagnetic studies in a finite element analysis tool. To develop an optimal, compact, and portable NMR tool, the magnetic pulling force must be optimized by considering the acceptable magnetic strength. For a comprehensive study regarding the force on a magnetic dipole and differences in pulling forces that are experienced by dipoles see Boyer (1998). Another main challenge to develop a portable NMR device is the homogeneity of magnetic arrays. The magnetic field homogeneity would be at the highest level when the mechanical structure of the NMR has an infinite length. However, in real conditions having a lengthy instrument is not practically feasible. In addition, as stated earlier, inaccuracy in the manufacturing process can effectively decrease the homogeneity. To overcome this challenge, magnetic arrangement for novel discrete Halbach layout approach is used in numerous designs processes (Soltner and Blümler, 2010). Thus, the final magnetic structure with limited length can have a homogenous field by placing small permanent magnets in an optimal location, which are found by running an optimization algorithm that mostly relies on analytical equations (Huber et al., 2018). Moreover, the mechanical strength of these structures should be considered in the fabricating process. When the number of the magnetic segments increases, the number of points that are vulnerable to be separated are increased as well. This is due to multi-direction pulling forces that are mostly effective between these Halbach magnets. This condition becomes more crucial when the NMR becomes more compact. It should be mentioned that by decreasing the number of magnetic parts in the structure, the mechanical strength of the design is upgraded noticeably at the cost of less homogenous magnetic field.

### Optimum dimensions of the test region

Determining the optimum dimension for the region of the test is another challenge in portable NMR devices (Klein et al., 2021). The air gap length can alter the homogeneity level of the magnetic field in the test region. Decreasing the airgap in the test region would improve the homogeneity level. However, this can constrain the practical development of the NMR tool from two points of view: first, the pulling force is increased drastically, and second, there would not be enough effective space for doing specific tests which need a holder around the understudy stem.

### Imaging and velocimetry challenges

Regarding the imaging and velocimetry applications in NMR devices, having a well-designed radio frequency (RF) coil is important to apply effective exciting signals on the understudy sample. Furthermore, in some NMR tools a receiving coil needs to be designed with high accuracy to observe the transition in nuclear spin. Generally, a static field is generated by a mechanical magnetic module, and if this structure is openable, a misalignment may occur in the structure by repeating the test, which is mainly because of the high amount of pulling forces. Thus, designing a robust openable structure to have an accurate imaging is crucial.

RF generator, amplifier, and spectrometers are required devices used to imaging and measuring velocity. The level of accuracy for generating pulses and receiving transitions in nuclear spin have significant impact. If the exciting signals have some deviations from their defined positions or the signal quality cannot not satisfy the minimum imaging constraints, the signal to noise ratio might have a lower value. Noted that, due to the high sensitivity of the high-frequency transmitter and receiver coils to the surrounding area, it is recommended that the test is carried out under conditions where external interfering disturbances are minimal. Certainly, satisfying these constraints to obtain a high-resolution image has numerous electromagnetic challenges. Addressing such constraints enables realization of an effective portable NMR tool for sap flow measurement. Despite such challenges, some research groups have succeeded in making compact and portable NMR tool so that experiments can be conducted *in situ*(climate chambers, greenhouses, or the natural environment) rather than transporting plants to the lab (Musse et al., 2017). In 1998, a portable NMR device named NMR-MOUSE (**Figure 4iii**) was constructed by Blümich et al. (1998) and used for a variety of applications, such as analysis of porosity of the materials (Blümich et al., 2010), stratigraphy of paintings in a non-destructive and non-invasive manner (di Tullio et al., 2016), leaf water status (Capitani et al., 2009), and moisture fraction in wood (Casieri et al., 2004). In a recent study, NMR-MOUSE was used to analyze the circadian variation of root water status in three herbaceous species (Nuixe et al., 2021). A further step in developing portable NMR is the use of permanent C-shape magnets (Rokitta et al., 2000). This type of magnet has good homogeneity and field strength, although it depends on the field strength and air gap. Nonetheless, its heavy weight limits its usefulness as a portable NMR device. Another study developed a Halbach-type permanent magnet arrangement (**Figure 4ii**) that could be opened from one side and closed around a target object: the NMRCUFF (for Cut open, Uniform, Force Free) (Windt et al., 2011; Blümler, 2016). Further this NMR CUFF based portable NMR was used to measure xylem sap flow in a poplar tree (Populus nigra) (Windt and Blümler, 2015). However, it is still a long way off from taking this portable NMR technology to the field and measuring sap flow in real-time. One of the recent attempts is made by Windt et al. (2021) in which all components are mounted including the magnet and spectrometer (with a total weight of 45kg) on a handled trolley and transported to the field. An imaging system weighing 45kg was used to measure quantitative water content in apple orchards (Meixner et al., 2021).

## Concluding remarks

**Figure 5:**
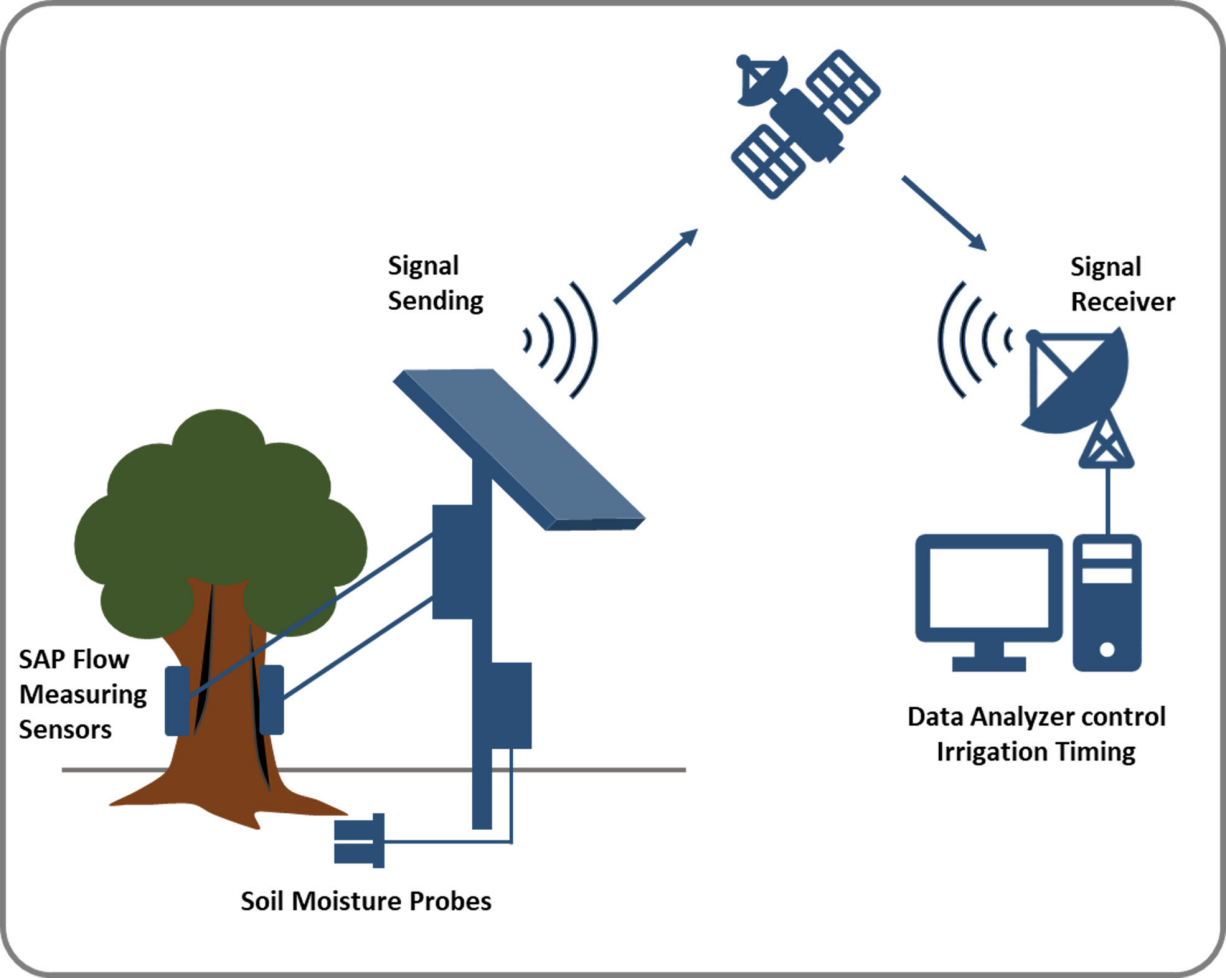
Model for monitoring the water content of plants using NMR combined with remote sensing tools for irrigation time management. In, future we need a portable NMR which can be used in the field and can be remotely controlled and remotely receive the data for the analysis.

Compact and portable NMR can be a potential solution as a powerful tool for gauging water movement in plants non-invasively. A non-invasive, portable, inexpensive NMR methodology for monitoring changes in plant water status that can be used in field conditions would greatly improve the understanding of vegetation responses and environmental feedback. Monitoring sap flow continuously at short time intervals is possible using NMR, which can help manage irrigation water. In future, new remote sensing tools and products with portable NMR can provide the best opportunities for measuring and modelling ecosystem-scale water fluxes (**Figure 5**). Moreover, there is a need for a global database where individual datasets containing sap flow data provided by contributors worldwide can be stored, analyzed, and used to develop prediction models based on which different models based on sustainable agriculture plants and water availability can be predicted which can be helpful in future changing climate condition.

Considering future climate change, the availability of an NMR sensor is critical for plant health and irrigation management. It is still challenging to measure sap flow using NMR outside a strictly controlled environment. However, with the intense research going on in developing portable NMR, in the near future we expect to use this technology to monitor sap flow in plants and trees non-invasively in the field.

## Author contributions

The first draft of the manuscript was written by RK, MH, and NH. MS, SJ, and BG revised the manuscript. All authors read and approved the final manuscript. All authors contributed to the article and approved the submitted version.

## Funding

This research was supported by the US National Science Foundation (NSF) through the Plant Genome Research Program (PGRP). The authors acknowledge the NSF for financial support through grant no. 1936376.

## Acknowledgments

The authors are grateful to Gerard Kluitenberg, Kansas State University, and Carel Windt, Forschungszentrum Jülich, for insightful discussions.

## Conflict of interest

The authors declare that the research was conducted in the absence of any commercial or financial relationships that could be construed as a potential conflict of interest.

## Publisher’s note

All claims expressed in this article are solely those of the authors and do not necessarily represent those of their affiliated organizations, or those of the publisher, the editors and the reviewers. Any product that may be evaluated in this article, or claim that may be made by its manufacturer, is not guaranteed or endorsed by the publisher.

## Abbreviations

CHPM, Compensation heat pulse; FAO, Food and agriculture organization; HFD, Heat field deformation; HPV, Heat pulse velocity; HR,Heat ratio; MRI, Magnetic resonance imaging; NMR, Nuclear magnetic resonance; SHB, Stem heat balance; TD, Thermal dissipation; TSHB, Trunk sector heat balance; TTD, Transient thermal dissipation.
